# Local Fractal Connections to Characterize the Spatial Processes of Deforestation in the Ecuadorian Amazon

**DOI:** 10.3390/e23060748

**Published:** 2021-06-14

**Authors:** Andrea Urgilez-Clavijo, David Andrés Rivas-Tabares, Juan José Martín-Sotoca, Ana María Tarquis Alfonso

**Affiliations:** 1 CEIGRAM, Centro de Estudios e Investigación para la Gestión de Riesgos Agrarios y Medioambientales, Universidad Politécnica de Madrid, 28040 Madrid, Spain; davidandres.rivas@upm.es (D.A.R.-T.); juan.martin.sotoca@upm.es (J.J.M.-S.); anamaria.tarquis@upm.es (A.M.T.A.); 2 Complex Systems Group, Universidad Politécnica de Madrid, 28040 Madrid, Spain; 3 IERSE, Instituto de Estudios de Régimen Seccional del Ecuador, Universidad del Azuay, 010204 Cuenca, Ecuador; 4 Departamento de Matemática Aplicada a las TIC, Universidad Politécnica de Madrid, 28040 Madrid, Spain

**Keywords:** Amazon basin, deforestation process, local connected fractal dimension, fractals, deforestation complexity

## Abstract

Deforestation by human activities is a common issue in Amazonian countries. This occurs at different spatial and temporal scales causing primary forest loss and land fragmentation issues. During the deforestation process as the forest loses connectivity, the deforested patches create new intricate connections, which in turn create complex networks. In this study, we analyzed the local connected fractal dimension (LCFD) of the deforestation process in the *Sumaco* Biosphere Reserve (SBR) with two segmentation methods, —CA-wavelet and K-means—to categorize the complexity of deforested patches’ connections and then relate these with the spatial processes. The results showed an agreement with both methods, in which LCFD values below 1 corresponded to isolated patches with simple shapes and those above 1 signified more complex and connected patches. From CA-wavelet a threshold of 1.57 was detected allowing us to identify and discern low and high land transformation, while the threshold for K-means was 1.61. Both values represent the region from which deforestation performs local aggressive expansion networks. The thresholds were used to map the LCFD in which all spatial processes were visually detected. However, the threshold of 1.6 ± 0.03 was more effective in discerning high land transformation. such as shrinkage and attrition, in the deforestation process in the SBR.

## 1. Introduction

Land transformation occurs as a result of natural processes or human activities affecting the landscape, from low to high levels. Human pressure has played an essential role in the land transformation of the Amazon basin tracing an unprecedented landscape footprint. Over the last four decades, exacerbated deforestation rates in Amazon have been increasing without declining [1,2,3,4]. Consequently, an increase in landslides because of rising erosion rates, floods resulting from change to the hydrologic regime, and climate variability affecting microclimate, among others are the main drivers of Amazon land transformation and ecosystem services loss. 

The loss of primary forest is a significant issue for Amazonian countries, such as Ecuador, Colombia, Venezuela, Peru, Bolivia, Guyana, Surinam, and Brazil. These countries have to face economic dilemmas from natural resource exploitation such as mining, oil, timber extraction, and agricultural expansion [5]. These were major and visible forest decline factors in the headwaters of the Amazon River basin [6,7,8]. Recently, since the 2000s agricultural expansion has been increasing spatially [9,10], becoming of great importance in the Ecuadorian Amazon, the Orinoquia region of Colombia, and Venezuela; this expansion process is strengthening as a belt that advances towards the Amazon lowlands [3]. This conversion of tropical forests into pastures and agricultural land evolves silently over time, causing changes in the environment and regional and local consequences.

Deforestation is a process with complex, diverse, and spatially variable features. This process occurs at different spatial and temporal scales and follows well-known spatial processes such as perforation, dissection, fragmentation, shrinkage, and attrition [11]. In the Amazon basin, the spatial processes vary from the basin headwaters to Brazil’s floodplains because of human activities such as unplanned villages, unauthorized roads, illegal mining pits, river blockages, drug trafficking, etc. Thus, the ecosystems have fragmented, causing animal connectivity loss, leading to a worrisome habitat modification with increases the unnatural forest edges as a result of the dynamics of plot fencing.

One highlighted case of primary forest fragmentation is the Ecuadorian Amazon which registered a natural forest loss of 19,000 km^2^ from 2000 to 2008 [12]. This loss was linked to ecological, social, economic, and cultural aspects at different scales [13], of which poverty was one of the leading causes [14]. The lack of policies and economic support in developing countries such as Ecuador contributes to the advance and strengthening of deforestation by creating new spatial connections between older and new deforested patches, causing a cumulative and complex effect of the spatial processes over time.

Despite spatial connections of deforestation being dynamic and challenging to measure quantitatively, they have been captured through LULC mapping at different spatial and temporal scales. In addition to allowing the monitoring, analysis, and documentation of land transformations, these maps have allowed the identification of geometric forms of these transformations at the naked eye, suggesting the existence of complex spatial patterns that can be studied not from conventional geometry but from fractal geometry.

Studies on the spatial connections of deforestation have been performed using the fractal dimension concept to describe the deforestation process’s overall behavior [15,16,17]. However, it is often difficult to discriminate and describe the spatial processes of deforestation patterns quantitatively, since these processes include the neighborhood’s influence on the pattern and how it evolves. Thus, we tested the potential of the fractal analysis, including the neighborhood concept to: (1) characterize and map the complex connections of the deforestation process and (2) categorize these connections based on spatial relationships for a cumulative period. As far as we know, this is the first time that fractal analysis has been used to characterize forest loss relations using non-linear geometry features associated with land spatial processes through the concept of local connected fractal dimension (LCFD). This approach has proven to be of great interest in various studies such as dynamics of forest fragmentation [15] and forest ecosystems [18,19], urban connectivity [20], fish gills analysis [21], etc. In this research, we coupled the fractal analysis through LCFD with landscape ecology concepts for studying the connections between cumulative deforested patches identified from LULC maps in the *Sumaco* Biosphere Reserve—(SBR) located in the Ecuadorian Amazon.

## 2. Materials and Methods

### 2.1. Study Area

**Figure 1:**
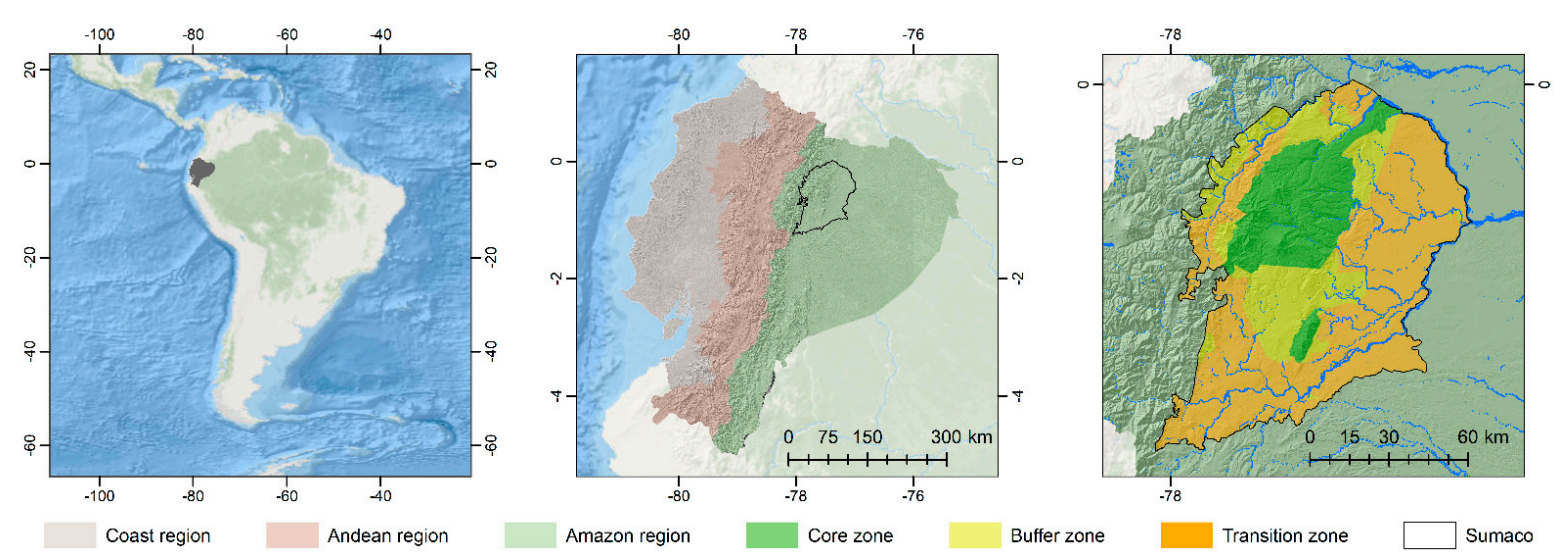
*Sumaco* Biosphere Reserve (SBR), located in the forest Amazon region of Ecuador.

The *Sumaco* Biosphere Reserve (SBR) was declared by UNESCO in 2000. It is located 100 km southeast of Quito, in western Napo province covering various Andean and tropical ecosystems. The SBR’s LULC includes natural forest, disturbed forest, shrub vegetation, herbaceous vegetation, grasses, and crops. The core zone mainly includes non-intervention forests and protected areas. The buffer zone contains representative examples of the country’s tropical rainforest and the Amazon region. The transition zone has intensive agricultural land use, Figure 1.

The SBR is in the Ecuadorian Amazon which corresponds to the forest region of Ecuador. This region is one of the most critical high-lands of the Amazon River basin, presenting the highest volcanos, such as *Tungurahua, Cotopaxi Cayambe, Chimborazo, Sangay,* and *Sumaco,* among others. All these volcanos providing enormous hydrologic regulation services for the Amazon River basin. This area also contains well-preserved habitat corridors and highly diverse fauna with enormous value for world biodiversity.

The Ecuadorian Amazon is the region in which most of the deforestation has been performed in Ecuador during the past few decades. However, this region also preserves extensive, intact, tropical moist forests that are endangered by colonists and today are hotspots for conservation purposes. 

### 2.2. Data and Processing

LULC maps from 1985 to 2018 from the MapBiomas project, collection 4.0 [22,23] were, used to perform spatio-temporal analysis of the deforestation process in SBR. These were in raster format with a spatial resolution of 30 m, and belonged to five categories: forest, non-forest natural formations, agricultural land, non-vegetation areas, and water. The MapBiomas project covers the Amazon biome and is fully automated and integrated into the Google Earth Engine (GEE) platform [24]. We use the GEE to extract LULC maps for the Ecuadorian Amazon (https://mapbiomas.org/ (accessed on 17 June 2020)). Since MapBiomas offers annually products from 1985 to 2018, we performed a deforestation process analysis every 5-years, except for the last map. We used the LULC maps of 1985, 1990, 2000, 2005, 2010, 2015, and 2018.

**Figure 2:**
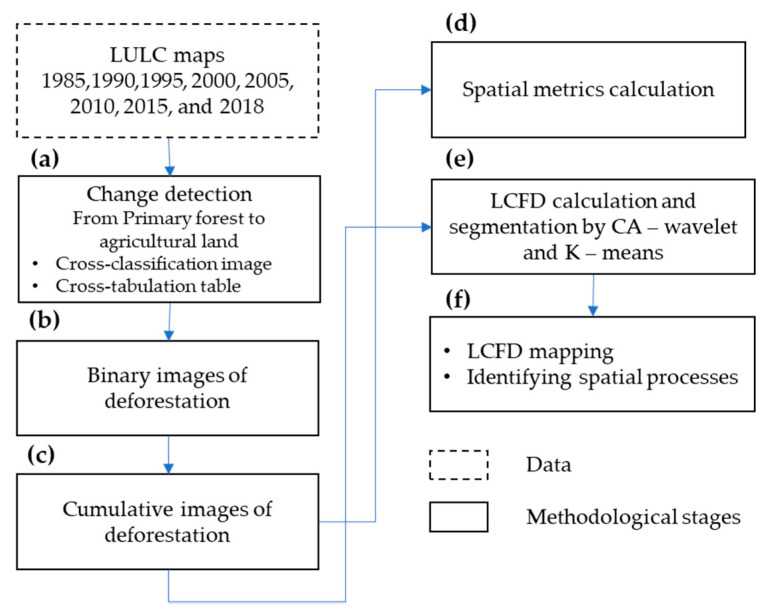
Methodological framework to characterize the deforestation process through local connected fractal dimension (LCFD) and spatial processes in the *Sumaco* Biosphere Reserve (SBR).

Using these maps, we detected LULC transitions using cross-classification images and cross-tabulation tables with R software, by applying the overlay and crosstab functions of the *Raster* package [25]. The first returned a map where the transitions were spatially located, and the second returned a table displaying the frequency distribution of LULC transition categories in each period: 1985–1990, 1990–1995, 1995–2000, 2000–2005, 2005–2010, 2010–2015, and 2015–2018. This study focuses on transitions from primary forest to agricultural land as the significant change (Figure 2a). Henceforth, in this work, this transition is referred to as deforestation.

LULC map transitions identified as deforestation were converted into binary images using the reclassify function of the Raster package. These transitions were classified as 0 (white) indicating primary forest loss in SBR and background with 1 (black) (Figure 2b). In this step, we obtained seven binary images—one for each period. Then, these images were accumulated, obtaining six binary images of the deforestation process: from 1985– 1995, 1985–2000, 1985–2005, 1985–2010, 1985–2015, and 1985–2018 (Figure 2c). The extent of these images was 4027 × 4669 pixels. 

### 2.3. Spatial Metrics and Spatial Processes of Deforested Patches 

**Figure 3:**
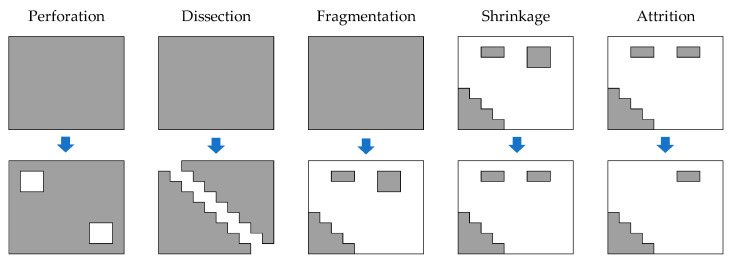
Graphical representation of the spatial processes in deforestation. The first row indicates the initial forest state and the second indicates the spatial processes. Adapted from [11].

We used the cumulative binary images of the deforestation to assess its behavior over time (Figure 2c) and calculated the deforested area and ratio in each cumulative time period. Morover, some other spatial metrics were calculated at patch level (Figure 2d), including patch density (PD), the number of patches (NP), edge density (ED), Euclidean-nearest-neighbor mean distance (ENN_MN), and clumpiness index (CLUMPY) (Table 1). These metrics were selected because of their importance concerning the spatial processes in land transformation [11] (Figure 3).

Two of the five spatial processes are defined as low-impact land transformation: (1) perforation, which is related to the dispersed agricultural plots, houses, and orchards of colonists inside the primary forest patches, and (2) dissection, which is a process characterized by spatial objects such as roads, power lines, irrigation infrastructure, and open channels, among another objects that dissect primary forest, with a constant width of the deforested strip. The third spatial process—fragmentation— is a spatial breaking point that differentiates the low-impact from the high-impact land transformation: shrinkage and attrition. Fragmentation is a critical process for landscape mosaics’ configuration. When fragmentation is reached, the subsequent forest loss is susceptible, and the connectivity between patches is fragile. Shrinkage is when the size of the remaining primary forest decreases, but patches can remain stable without disappearing. Attrition is the last stage, in which the primary forest patch disappears. 

### 2.4. LCFD Calculation of the Deforestation Process

**Figure 4:**
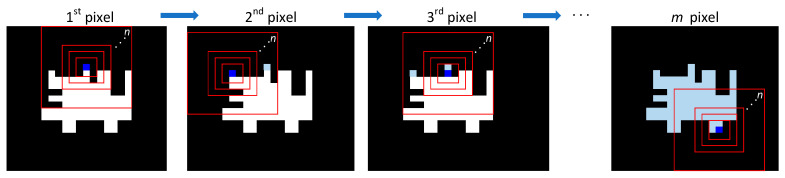
Scanning pixel procedure to calculate the local connected fractal dimension (LCFD). White pixels represent deforested patches, pixels in bold blue represent the currently scanned pixel, and the light blue pixels represent those scanned, from the first to the *m^th^* pixel. Red boxes indicate the scanning extent by *n* box sizes.

Using cumulative binary images of the deforestation process, we calculated the local connected fractal dimension (LCFD) [43], taking advantage of the FracLac tool [44] of ImageJ software [45] (Figure 2e). Figure 4 shows the scanning image procedure, which starts sampling every deforested pixel (in bold blue) from the upper left corner to the right, and then to the bottom (pixel in light blue). Each scan step included a rigorous zoomed-out scan of the pixel neighborhood, calculating the LCFD in each box size (red box in Figure 4). Scanning only considers the pixels within the largest box that belongs to the cluster connected to the pixel on which the box is centered [20].

This method was applied to all of the deforested pixels of each cumulative binary image. The LCFD was calculated by the linear regression of the logarithm of the mass (pixels), M(ε) in a box of size ε on the logarithm of ε. This scaling relation is expressed in the following equation:

(1) $$LCFD=\frac{Log\;\left[M\left(\varepsilon \right)\right]}{Log\;\varepsilon }$$

where M(ε) is the number of locally connected pixels (eight-neighborhood connection) in a box of side size ε [43]. From equation 1, the LCFD = 2 when the object is completely filled, and therefore the object is two-dimensional. On the other hand, if the object is a straight line (one-dimensional), then the LCFD = 1. The LCFD results are more useful for values in the range 1.0 < LCFD ≤ 2.00, because these describe the local complexity of the set. Although the former concept of LCFD refers to how the fractal fills the space, the fractal dimension of forest loss can be understood as a measure of “emptying” and not “filling”, as the traditional concept states. From this point of view, an LCFD close to 2 for forest loss analysis measures the lack of forest, which implies that other land uses, such as agriculture and grassland, can occupy these gaps.

Previous work in the SBR showed that the deforestation process exhibited lacunar scaling properties, achieving a minimum box size length of 46 pixels [46]. We chose the maximum limit of this box size length in order to keep the analysis at a local scale. Another work, aiming to characterize local retinal vessel abnormalities, used 31 pixels as the maximum local connected set size [43]. Therefore, in this study, the range of the scales selected for the locally connected set was from 3 to 31 pixels with a 2-pixel increment step, giving a total of 8 box sizes (*n*); that is, box side lengths of 3, 7, 11, 15, 19, 23, 27, and 31 pixels. That means a side length range from 90 to 930 m. During the scanning process, the box slid one pixel both horizontally and vertically.

Once the entire image was scanned, we obtained two results: (1) LCFD frequency distribution, and (2) an LCFD text file with LCFD values for each pixel as (X, Y) list. These were used to define thresholds, map the local connections, and identify spatial processes.

### 2.5. LCFD Thresholding and Mapping

**Figure 5:**
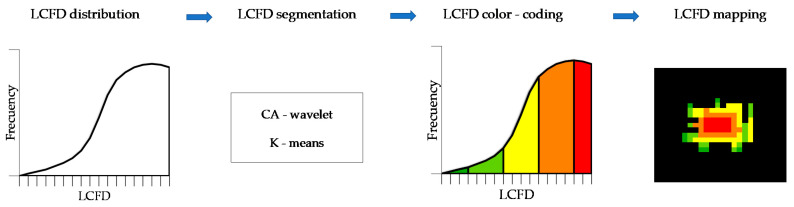
The local connected fractal dimension (LCFD) segmentation procedure and mapping.

Figure 5 shows a procedure for segmentation and mapping of LCFD (Figure 2e,f). LCFD frequency distribution was used to define several LCFD categories or thresholds, which assist in characterizing the spatial processes of deforestation by coloring the LCFD into maps. To set LCFD thresholds we use two methods: CA-wavelet, and K-means. Both present reliable and satisfactory procedures to classify spatial structural patterns. CA-wavelet was used in other research for segmenting soil images using fractal features [47,48], and K-means was used similarly to discern urban sprawl patterns [49] and vessel complexity in patients with cerebral arteriovenous malformations [50], to mention some diverse application cases.

Mapping of the LCFD was performed from the LCFD text file. This file was converted into a georeferenced LCFD raster with a pixel resolution of 30 m using R software. This raster is a grayscale image that contains LCFD values in each pixel. Based on thresholds defined by CA-wavelet and K-means, we classified and colored the image in order to characterize the connectivity of deforestation as a network of the LCFD, as shown in the last panel of Figure 5.

#### 2.5.1. Concentration-Area (CA) and Wavelet -Transform Modulus -Maxima (WTMM) Methods

Fractals have the property of self-similarity, in which the spatial variability of the studied variable is scale-independent. Therefore, fractal models commonly result in power-law relationships between the variables of interest.

The CA method [51] establishes power-law relationships between the concentration of a variable and the area enclosed by this concentration:

(2) $$A\left(\rho \geq C\right)\propto C^{\beta }$$

where A is the area constituted by concentrations (ρ-values) greater than a given value C and β is the characteristic exponent of the CA method.

When the studied variable’s spatial variability follows a fractal model, this power-law relation has only one exponent. The CA method occasionally does not meet the simple fractal model, because different power laws apply to the studied variable. In this case, we can assume that the spatial variability is multifractal instead of fractal. Thus, different slope-change values in the log-log plot can be considered to be separations among different sets of the variable.

The continuous wavelet transform (CWT) of a function $f\left(t\right)\in L^{2}\left(\mathbb{R}\right)$ is a space–scale transformation defined by the inner product:

(3) $$Wf\left(u,s\right)=f,\psi_{u,s}=\int_{-\infty }^{\infty }f\left(t\right)\frac{1}{\sqrt{s}}\psi^{*}\left(\frac{t-u}{s}\right)dt$$

where t,u∈ℝ and $s\;\left(scale\right)\in {\mathbb{R}}^{+}$. The family of functions $\psi_{u,s}\left(t\right)$ ∈ $L^{2}$(ℝ) is called “wavelets”, which are scaled and translated versions of a particular function ψ(t). The function ψ(t) is called the “mother wavelet”:

(4) $$Wf\left(u,s\right)=f,\psi_{u,s}=\int_{-\infty }^{\infty }f\left(t\right)\frac{1}{\sqrt{s}}\psi^{*}\left(\frac{t-u}{s}\right)dt$$

ψ(t) often satisfies the zero-mean and fast-decay properties, assuring the existence of the Inverse CWT (ICWT).

In order to study the local regularity of a function to find singular points, it is necessary to use a wavelet with vanishing moments, i.e., ψ(t) must be orthogonal to n-order polynomials. In this case, the CWT can be interpreted as a multiscale differential operator of order n. Mallat’s theory [52] establishes that the CWT redundancy can be eliminated only by using the WTMM method to detect singular points in a function. These maxima are defined as the local maxima of |Wf(u, s)| at each scale s and are located on connected lines in the space–scale plane. Thus, these lines are named “maxima lines”. An important characteristic of these maxima lines, in the context of singularity detection, is that at least one maximum line converges toward each singular point.

The WTMM method has been demonstrated to be an effective method for locating singular points in 1D and 2D functions [53]. If the wavelet has only one vanishing moment, these maxima lines are used to locate discontinuities associated with edges in images. If the wavelet has two vanishing moments —e.g., the Mexican hat wavelet— then the maxima lines converge toward maximum curvature points. In this study, we applied CA-wavelet to LCFD list values for the whole cumulative period 1985–2018.

#### 2.5.2. K-Means

Since the LCFD is calculated based on its relationship with the neighborhood from the box-counting sampling, clustering the LCFD is desirable in order to map deforestation complexity. The K-means algorithm [54] was used to segment LCFD grayscale images and to identify and visualize the five main spatial processes in deforestation using a fractal approach. This algorithm is one of the most valuable and reliable methods for unsupervised classification, highlighted by its simplicity when using a relatively low number of iterations.

K-means was also implemented to segment LCFD images, because of the high spatial correlation performance of the algorithm, and to minimize the group differences from the cluster centroid. Previous to performing the K-means, the NbClust package in R [55] was used to determine the optimal number of clusters to initialize the K-means. The K-means was applied over the LCFD map in QGIS using the Semi-Automatic Classification Plugin [56]. The algorithm rule used 0.001 as the minimum distance between classes, and 20 iterations were performed for the grayscale LCFD images from 1985 to 2018. From the resulting classes, minimum and maximum values per class were extracted in order to define LCFD thresholds. These thresholds were selected and applied for the other cumulative images.

## 3. Results

### 3.1. Cumulative Evolution of Deforestation

**Figure 6:**
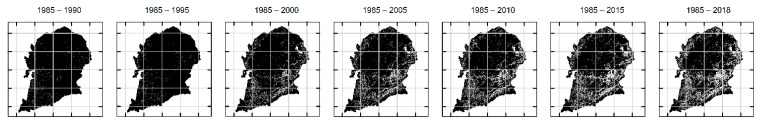
Cumulative binary images of the deforestation process in the *Sumaco* Biosphere Reserve (SBR) from 1985 to 2018. White pixels represent the deforested patches.

Figure 6 shows an increase in deforestation from 1985 to 2018. The accelerated deforestation process mainly occurred during the period 1995–2000, because of agricultural expansion. This time point was well known in Ecuador as the “inclusive agriculture” and “inclusive business” programs period, supported by public and private institutions [57]. The forest loss was accentuated for this period in the southeast and southwest of the transition zone of the SBR. The primary steps of deforestation from 1985 to 2000 settled the base of deforestation expansion until 2018. This result suggests that the clearances from the primary forest in the SBR strengthened the process from the initial patches, and then developed, following spatial processes over time. The cumulative deforestation, from 1985 to 2005, started to define some visible paths at the SBR. In 2010 these paths started to connect deforested areas from the east to the west. This process indicates that white pixels started to promote an isolation structure of black patches, especially around the core area in the southern part of the SBR. The evolution until 2015 started to define a dendritic structure well defined and strengthened in dense black spots in the southeastern part of the SBR. Until 2018 deforested patches showed high connections extended throughout the biosphere reserve.

Spatial metrics of the deforestation process by cumulative periods are shown in Table 2. There is a clear breaking point in deforestation rates in the third cumulative period, increasing the DA 5.65 times in only 5-years. This fact can also be reflected in the NP, indicating that the DA increases around old deforested patches and fosters new clearances inside the primary forest patches. Then, the NP started to decrease at the same time that the DA increased until 2018. This indicates that deforestation was consolidated, and the patches developed by 2000 started to grow until 2018. The PD showed a peak during the period 1995–2000 and then the PD decreased until 2018. This is similar to the NP, since the PD is the number of patches per km^2^, and both are correlated. The ED in the SBR increased until 2015, then in 2018 decreased when connections between the forest patches started to disappear. This was because of the removal of residual, isolated primary forest patches and the consolidation of more homogeneous patches of deforested land. The larger deforested patches from 2015 to 2018 were characterized by larger sizes and regular shapes, resulting in a diminution of the ED.

The ENN_MN metric in the SBR decreased over the entire period, while the DA increased. The ENN_MN presented a minimum in 2005; then, the metric started to increase again until 2018. A decrease in an ENN_MN local minimum can be understood as a point where the deforested area was consolidated in an extended fragmentation with an irreversible state. This is because of the increasing connections between deforested patches and the growth of these. The last metric, CLUMPY, increased throughout the periods. This shows that the deforested patches were clumped, consolidating the deforestation process. CLUMPY showed a significant increase from 1995 to 2000 and 2015 to 2018, this was around a 5% in both periods, suggesting a high contagion level.

### 3.2. The Evolution of Local Fractal Connections

**Figure 7:**
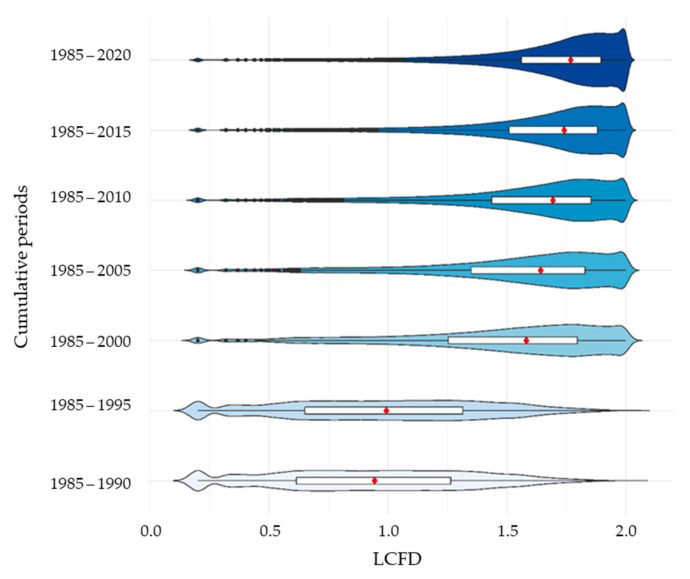
The local connected fractal dimension (LCFD) distribution of the deforestation process in the *Sumaco* Biosphere Reserve (SBR) by cumulative periods from 1985 to 2018.

The temporal evolution of LCFD distribution for each of the seven images is shown in the violins of Figure 7. The most illuminating period is the evolution between 1995 and 2000, in which the LCFD median increased from 0.94 to 1.57. From 2000, the LCFD increased until 2018, when the median reached 1.78. The violins show that the frequency distribution was negatively skewed. This suggests an increase in the deforestation process’ complexity over time, regarding the initial primary forest of 1985. LCFD frequency distribution tends toward a skewed effect because of cumulative deforested patches throughout the periods. The skewed effect is to be expected in violin graphs, in which the deforested area increases over time. From 2000, LCFD distribution was skewed to the left with higher values of LCFD, while at the same time, the NP decreased, and the total deforested area increased (Table 2). This state of the deforestation process suggests a consolidation of forest loss with a high connectivity network with complex spatial features. 

### 3.3. LCFD Thresholds with CA-Wavelet and K-Means

The spatial processes’ connections start with perforation and dissection of the pri-mary forest, but this remains relatively important at the regional scale. Simultaneously, fragmentation and shrinkage settle deforestation issues at the landscape level, and at-trition is when forest patches disappear completely. In this case, the LCFD and deforestation spatial processes were matched using segmentation algorithms. The LCFD was calculated for each cumulative image of the SBR, and the results subsequently segmented using the CA-wavelet and K-means methods.

**Figure 8:**
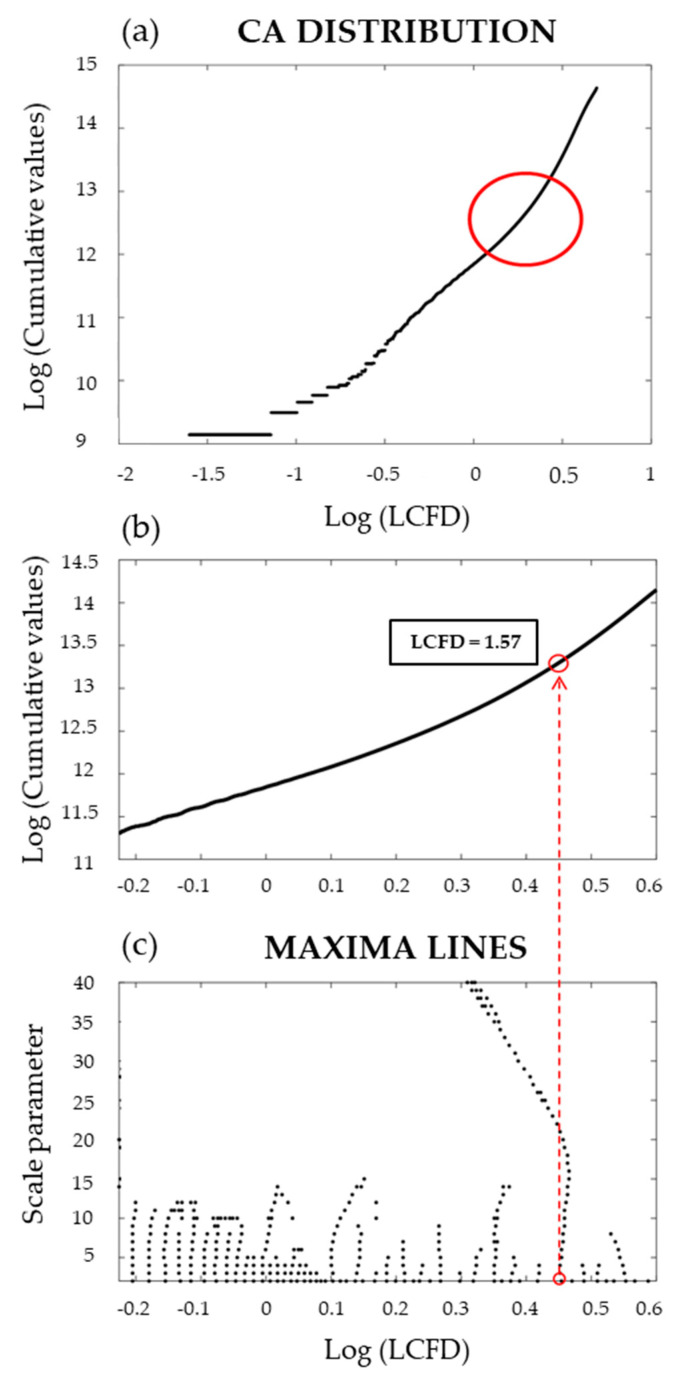
CA-wavelet singular point identification in the local connected fractal dimension (LCFD) segmentation.

For LCFD segmentation with CA-wavelet (using a Mexican hat wavelet), the results are shown in Figure 8. In this figure, a singular point was identified in the CA distribution of the LCFD, corresponding to the slope-change point marked with a red circle in Figure 8a. Then, the WTMM method was applied in order to locate this anomaly in the CA distribution. The points where the maxima lines converge denote the locations of anomalies in the curvature, Figure 8c. The slope-change point was identified corresponding to the LCFD of 1.57 (Figure 8b). This singular point suggests a partitioning threshold to segment LCFD distribution in the SBR.

**Figure 9:**
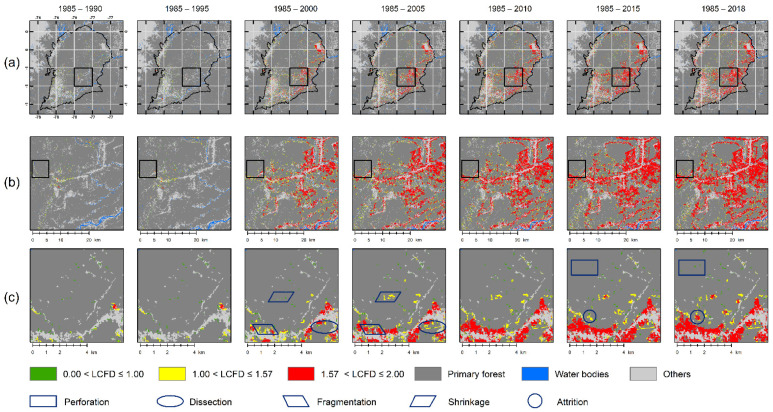
The local connected fractal dimension (LCFD) thresholds of deforestation of the deforestation process in the *Sumaco* Biosphere Reserve (SBR) from 1985 to 2018, using CA-wavelet. The first row (**a**) is a complete view, the second row (**b**) is an enlarged view of (**a**), and the third row (**c**) is an enlarged view of (**b**). Shapes in dark blue are the spatial processes identified.

Figure 9 shows the colored LCFD map results when applying the CA-wavelet threshold. In this, thresholds of 1.0 and 1.57 were used. The CA-wavelet method did not detect maxima lines around the value of 1.0. However, a threshold 1.0 was used in this segmentation because of its geometrical meaning: it separates unconnected points into one-dimensional clusters. This can be observed in the LCFD progress, from 1985 to 2018, in row B. Analyzing these zoomed-in squares reveals that the connections have progressed to a complex mosaic. The second zoomed-in squared in row C, indicates at the local scale the five spatial deforestation processes (dark blue outline boxes) and the relationships between them with the color map of the LCFD. Even though these processes are intricate in space at different scales, we selected samples for better understanding than the graphical interpretation of spatial deforestation processes presented in Figure 3.

**Figure 10:**
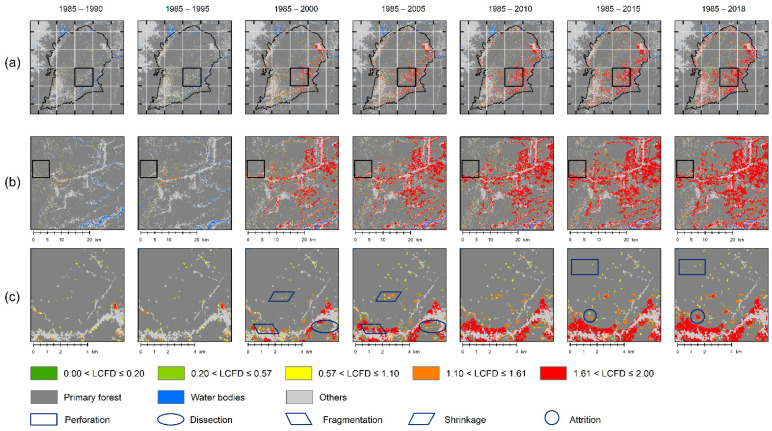
The local connected fractal dimension (LCFD) thresholds of the deforestation process in the *Sumaco* Biosphere Reserve (SBR) from 1985 to 2018, using K-means. The first row (**a**), is a complete view, the second row (**b**) is an enlarged view of (**a**), and the third row (**c**) is an enlarged view of (**b**). Shapes in dark blue are the spatial processes identified.

The results for the LCFD threshold of K-means are shown in Figure 10. The optimal number of clusters for K – means was five classes using the NbClust algorithm. The re-sulting thresholds were 0.2, 0.57, 1.10, and 1.61. These come from the clustering minimum and maximum values of the classes, as Figure 9 and rows B and C of Figure 10 show zoomed-in areas where local connections show progress over time, and result in a very fragmented mosaic of the primary forest in the SBR by 2018. Despite the two algorithms being different, the thresholds were very close to 1.0 and 1.6, suggesting that these singular points of the LCFD offer the most significant connection characteristic of the deforestation process from a fractal approach.

Similarly, in Figure 10, with K-means segmentation, the spatial processes were identified in dark blue outline boxes. Within these boxes, it is clear to note that classes 4 and 5 correspond to orange and red LCFD clusters, and these represent shrinkage and attrition processes, respectively. While perforation, dissection, and fragmentation were more challenging to discern from cluster-colored coding all three processes were at least under 1.1 LCFD values. Thus, CA-wavelet rightly groups low-impact forest loss stages under LCFD < 1.00 and high-impact forest loss stages higher than 1.57. K- means and CA-wavelet work together to define accurate thresholds of the local connections of fractal features of spatial deforestation processes.

## 4. Discussion

### 4.1. Major Spatial Attributes of Deforestation Processes

The deforestation process results from driving factors that interact in time and space, changing the landscape into a heterogeneous and complex system. Over the last 28 years, the SBR lost over 2100 km^2^ of its primary forest because of agricultural and livestock activities. This drove the SBR into a high-fragmentation landscape, affecting connectivity within protected and non-protected areas. The main spatial processes involved in this resulting landscape mosaic were fragmentation and shrinkage. The SBR is not the only site in which deforestation has affected the landscape configuration; other Ecuadorian biosphere reserves with similar characteristics and similar landscape fragmentations are Podocarpus –El Condor [58], and Bosque Seco [46].

In the SBR as time has passed the landscape composition has varied in terms of deforestation area dynamics since the NP has increased, fragmenting and isolating the primary forest over time. On the other hand, the landscape configuration through the ED showed a peak in 2015, and then slowed down up to 2018. This signifies that the patches have merged and increased their size as the NP decreased. Overall, the fragmentation of primary forest before 2010 starts to be dominated by high-impact spatial processes, such as shrinkage and attrition. The resulting landscape is to be expected when the deforestation process is uncontrolled despite this being a declared biosphere reserve area. A similar process was also detected in the state of Rondônia in the Brazilian Amazon with a total deforested area of 2300 km^2^ [27].

Once the primary forest fragmentation was identified, the ENN_MN metric was important to distinguish the isolation degree. In this case, the stepped diminution of the ENN_MN meant greater proximity between the deforested patches, facilitating their connection and strengthening the shrinkage and attrition processes. In terms of the temporal analysis of deforestation patches it is desirable that the ENN_MN over time be as higher as possible, in order to reduce the connectivity between deforestation patches that provokes isolation of primary forest patches.

CLUMPY is the purest landscape fragmentation index among the landscape metrics, since it is independent of patch abundance [59]. In the SBR, the CLUMPY was an important monitoring metric for the deforestation process in which lower values of around 0.4 are desirable for low-impact deforestation, and values that surpass 0.5 suggest continuous monitoring of deforestation spatial processes. The characteristics of this metric can be very useful as a strategy to follow at different scales for spatial planning and monitoring that would benefit forest connectivity, biodiversity conservation, and local livelihoods in different ways [60]. Thus, spatial metrics offer a classical view and a quantitative indicator of deforestation spatial processes elucidating the complex interaction of the factors behind deforestation [28].

### 4.2. Improvement of Fractal Characteristics through the Local Connections Approach

The deforestation process in the SBR presented a diversity of deforestation spatial processes, in which low-impact processes such as perforation and dissection occur at different scales. These processes showed low LCFD values (< 1.0) in which neighbor connections were not complex, because of the simple geometric significance of the fractal dimension in regular, dispersed, and small patches. Perforation and dissection were also characterized with low values for PN, ED, and CLUMPY, but high ENN_MN. Once the LCFD surpasses the threshold of 1.0, the fragmentation can be easily developed around these patches at different scales. From LCFD mapping, the color-coding for pixel values below 1.0 indicated low-impact forest loss with feeble connection, as shown in Figure 9 and Figure 10. When this condition is persistent over time, it means that the deforestation was controlled or in a static condition; these areas can be identified from mapping, and could be designated areas for periodic monitoring. K-means, used as a spatial algorithm, successfully detected these low-impact-forest-loss spatial processes with a fractal dimension below 1; this geometric representativeness meant low connectivity—something that is very clear from the fractal approach.

Some previous studies using fractal-connected features for spatial forest analysis also agreed that fractal dimensions below 1.0 were attributed to unconnected patches, while values above 1.0 corresponded to a mix of connectivity, forming irregularly shaped clusters [15,18]. However, these studies do not deepen the segmentation of fractal connection, and temporal LCFD quartiles were reported for administrative units. These studies were applied for forest fragmentation assessment using the LCFD, in which temporal analysis showed decreasing LCFD values. In the case of the SBR, the LCFD was applied for the assessment of deforestation patches, showing the increasing trend over time. Both deforestation and forest analysis via LCFD offer an understanding of forest fragmentation at a local scale as one of the main continental land transformation dynamics over in the past few decades.

The LCFD for deforestation was identified as the fractal capacity of every pixel in the image to trigger a network of the spread spatial processes. The local connections in the SBR indicated the complex relations of the fragmentation features in the landscape along stream networks, transportation, flatlands, transition, and buffer zones, favoring forest patch isolation from the core area. All of these represent local irregularities of the deforestation patches’ geometry. We have already reported that the deforestation process in the SBR was multifractal and multiscale, highlighting the spatial relationship with soil suitability [46]. Thus, the LCFD serves to complement the assessment of landscape connectivity to proximal objects at a municipal scale, such as routes, protected areas, ecological corridors, etc. We suggest including the LCFD to ensure the understanding of land transformation dynamics at the municipal level. Furthermore, the LCFD could serve as a metric to assess land-use planning programs, especially in areas that face accelerated changes in the primary forest.

The application of the LCFD was useful for characterize the entire image obtaining a set of values per pixel, while segmentation methods allowed us to establish thresholds by which to visually connect clusters of similar LCFD values. LCFD thresholds are very important for characterize spatial process issues at diverse scales and in different fields, such as medicine [43,61], urbanism [20], materials [62], etc. For example, in medicine, the LCFD threshold is useful identifying potential cancer expansion and local connections to proximal tissues. A threshold of 1.5 was identified for revealing very high intercellular connectivity to proximal tissues in patients who develop recurrence or metastases after cancer treatments, and low LCFD values indicate that local complexity in carcinomas is low [61]. In urban studies, a threshold of 1.5 is useful for characterizing urban connectivity and complexity. This threshold was used as the breaking point of a transition phase of the dynamic urban behavior that guarantees historical sites’ continuity, homogeneity, and coherence [20]. The former results constitute interpretation examples for thresholds’ usefulness in diverse fields. The LCFD in deforestation studies serves as novel complexity criteria by which to identify and discern low and high land transformation, especially to georeference local aggressive networks of deforestation processes—in this case assessing deforestation in the SBR.

Other works have reported general fractal analysis of deforestation in the Amazon region [16,26,63,64] and in Ecuador [46], but this is the first time that local fractal connections of deforestation process have been quantified and mapped in the Ecuadorian Amazon region. Something that is always requested for forest conservation programs is the provision of local solutions to focus on specific zones for restoration activities. Thus, LCFD mapping provides a feasible solution for decision-making at the local scale, since maps serve to interact with local stakeholders and to address specific implementation measures. However, mapping the LCFD in larger areas requires high-quality LULC maps with at least annual maps. The MapBiomas product for the Amazon biome provides high-spatial-resolution LULC maps for regional and national studies, but more effort is needed to improve their spatial accuracy and temporal resolution for local applications. The scarcity of LULC map sources with accurate spatial and temporal features can limit the application of LCFD mapping worldwide.

## 5. Conclusions

This is the first time LCFD mapping and segmentation methods have been used to evaluate deforestation spatial processes in the Amazon region. This approach provides a spatio-temporal procedure to assess spatial deforestation processes with accurate results, facilitating direct usage in a GIS (geographical information system) format essential to model land use and cover change in biodiverse world hotspots such as biosphere reserves. The main conclusions achieved in this work are:

- LCFD connections can be understood as a spatial index with which characterize the intricate connectivity of deforestation patterns.
- CA-wavelet and K-means show consistent segmentation algorithms for the LCFD of the deforestation process, which is essential for mapping interpretation of deforestation complexity in land management programs.
- LCFD mapping can be used to define spatial priority settings to tackle deforestation expansion in the Amazon region.
- This information can detect degradations hotspots based on complex relationships identified from LULC maps.

As an overall conclusion of this study, LCFD maps can help decision-makers to efficiently address financial items, knowing that these countries have low budgets for reforestation programs.

## Figures and Tables

**Table 1:** Description of the spatial metrics used in the characterization of deforested patches.

Spatial Metrics	Description	Unit	References *
DA	Deforested area: the sum of the areas of all deforested patches.	ha	[26,27,28,29]
Ratio	Ratio: the proportion between the actual (n) landscape class change with respect to time n-1.	proportion	[27]
NP	Number of patches: the number of patches for each landscape class.	Unit (N)	[28,30,31,32,33,34]
PD	Patch density: the density of the patches for each landscape class (number of patches per unit of area), representing an aspect of fragmentation—dissection of patches. Higher values represent a more fragmented landscape.	N/100 ha	[26,27,29,30,35]
ED	Edge density: the amount of edge relative to the total landscape area. This metric facilitates comparison at different extent sizes.	m	[26,29,32,35,36,37,38]
ENN_MN	Euclidean nearest neighbor mean distance: the mean distance between patches of the same landscape class, which could represent another aspect of fragmentation—connectivity between patches. Values range from 0 (adjacent patches) to infinity.	m	[27,28,30,38,39,40]
CLUMPY	Clumpiness index: measures the degree to which the landscape class is aggregated or clumped given its total area. This is the measure of patch aggregation. Values of the clumpiness index close to -1 are a measure of a maximally disaggregated landscape class, whereas values of the clumpiness index close to 0 are indicative of distributed random patches and when the clumpiness index approaches 1, the deforestation patch type is maximally aggregated.	none	[37,39,40,41,42]

* Authors using the given metric for the characterization of the deforestation process in the Amazon region.

**Table 2:** Spatial metrics of deforested patches in the *Sumaco* Biosphere Reserve (SBR) by cumulative periods from 1985 to 2018. CP: cumulative period; DA (ha): deforested area (ha); NP: number of patches; PD: patch density; ED: edge density; ENN_MN: euclidean-nearest-neighbor distance mean and CLUMPY: clumpiness index.

CP	DA [ha]	Ratio	NP	PD	ED	ENN_MN	CLUMPY
1985–1990	15,221	-	32,536	3.26	10.94	112.21	0.45
1985–1995	18,503	1.21	35,962	3.60	12.87	106.75	0.47
1985–2000	103,006	5.57	52,050	5.21	39.89	92.68	0.68
1985–2005	131,649	1.28	51,937	5.20	47.54	91.43	0.69
1985–2010	155,574	1.18	47,119	4.72	52.05	92.06	0.70
1985–2015	185,854	1.19	41,125	4.12	56.28	94.11	0.72
1985–2018	211,555	1.19	38,330	2.27	35.72	96.89	0.76

## Data Availability

The data presented in this study are available on request from the corresponding author. The data are not publicly available due to ethics and privacy.
